# Dosage-Dependent Expression Variation Suppressed on the *Drosophila* Male *X* Chromosome

**DOI:** 10.1534/g3.117.300400

**Published:** 2017-12-13

**Authors:** Hangnoh Lee, Dong-Yeon Cho, Damian Wojtowicz, Susan T. Harbison, Steven Russell, Brian Oliver, Teresa M. Przytycka

**Affiliations:** * Laboratory of Cellular and Developmental Biology, National Institute of Diabetes and Digestive and Kidney Diseases, Bethesda, Maryland 20892; † Computational Biology Branch, National Center for Biotechnology Information, National Library of Medicine, National Institutes of Health, Bethesda, Maryland 20894; ‡ Laboratory of Systems Genetics, National Heart, Lung, and Blood Institute, Bethesda, Maryland 20892; § Department of Genetics and Cambridge Systems Biology Centre, University of Cambridge, CB2 3EH, United Kingdom

**Keywords:** *Drosophila*, CNV, dosage compensation, expression noise, MOF, X chromosome, Genetics of Sex

## Abstract

DNA copy number variation is associated with many high phenotypic heterogeneity disorders. We systematically examined the impact of *Drosophila melanogaster* deletions on gene expression profiles to ask whether increased expression variability owing to reduced gene dose might underlie this phenotypic heterogeneity. Indeed, we found that one-dose genes have higher gene expression variability relative to two-dose genes. We then asked whether this increase in variability could be explained by intrinsic noise within cells due to stochastic biochemical events, or whether expression variability is due to extrinsic noise arising from more complex interactions. Our modeling showed that intrinsic gene expression noise averages at the organism level and thus cannot explain increased variation in one-dose gene expression. Interestingly, expression variability was related to the magnitude of expression compensation, suggesting that regulation, induced by gene dose reduction, is noisy. In a remarkable exception to this rule, the single *X* chromosome of males showed reduced expression variability, even compared with two-dose genes. Analysis of sex-transformed flies indicates that *X* expression variability is independent of the male differentiation program. Instead, we uncovered a correlation between occupancy of the chromatin-modifying protein encoded by *males absent on the first* (*mof*) and expression variability, linking noise suppression to the specialized *X* chromosome dosage compensation system. MOF occupancy on autosomes in both sexes also lowered transcriptional noise. Our results demonstrate that gene dose reduction can lead to heterogeneous responses, which are often noisy. This has implications for understanding gene network regulatory interactions and phenotypic heterogeneity. Additionally, chromatin modification appears to play a role in dampening transcriptional noise.

Wild-type alleles are overwhelmingly dominant to loss-of-function alleles (Wright 1929) because reducing gene activity by half does not result in halved activity from downstream genes. This has been elegantly demonstrated in *Drosophila*, where systematic analysis of segmental aneuploid viability showed conclusively that the dose of individual genes rarely has an overt phenotypic consequence, whereas altering the dose of many genes is lethal owing to additive effects (Lindsley *et al.* 1972). The organization of gene products into networks provides a high degree of passive robustness to reduced gene function (Kacser and Burns 1973, 1981; Orr 1991; Becskei and Serrano 2000). Part of this dampening of flux through a network is a physical property, due to the inescapable fact that gene activity is a series of biochemical events subject to kinetics (Coulon *et al.* 2014; Chow *et al.* 2015). This is also evident in *Drosophila*, where tissue culture cells show sublinear responses to gene dose (Zhang *et al.* 2010; Lee *et al.* 2014). Expression profiling of multilocus deletions (deficiencies; *Dfs*) shows that the primary effects of reduced transcription of one-dose genes coherently spread through the gene expression network and are ultimately absorbed (Malone *et al.* 2012; Lee *et al.* 2016), indicating that dose responses involve network interactions. Many studies focus on responses of gene expression levels to dose, but gene dose differences and network connections (Lander 2011) also contribute to expression variability, often referred to as expression noise.

Initial studies on gene expression noise focused on cells. Genetically identical cells, grown under identical conditions, exhibit quite pronounced gene expression diversity. At least some of these differences are due to stochastic events related to kinetics, such as transcriptional bursting (Kaern *et al.* 2005; Raser and O’Shea 2005; Raj and van Oudenaarden 2008). These mechanisms cannot easily explain the variation in organism-level gene expression (Elowitz *et al.* 2002; Swain *et al.* 2002; Raser and O’Shea 2005; Raj *et al.* 2006; Gibson 2008; Lin *et al.* 2016a). Stochastic events in single cells should average out when measurements are made in tissues or organisms. Thus, organism-level expression variability is more like to be mediated by evolved control mechanisms such as feedback modules, rather than the physics biochemical kinetics. These modules measure changes due to the environment, development, random mutations, or stochastic processes and make active adjustments (Becskei and Serrano 2000; Alon 2007; Malone *et al.* 2012; Wagner 2013; Lee *et al.* 2016).

Pioneering work on transcriptional noise at the single-cell level (Elowitz *et al.* 2002), and the first mathematical models of the process (Swain *et al.* 2002), separated sources of stochastic gene expression variation into intrinsic and extrinsic noise. Although the ultimate source of expression variability is difficult to trace, the formal definitions of intrinsic and extrinsic noise are valuable constructs for thinking about both passive buffering and active regulation. From a gene-centric point of view, intrinsic contributions to noise result from the biochemical stochasticity of kinetics, resulting in gene dose-sensitive transcriptional bursting, for example (Elowitz *et al.* 2002; Swain *et al.* 2002; Raser and O’Shea 2005; Raj *et al.* 2006; Salari *et al.* 2012). Such intrinsic gene expression noise is traditionally modeled using the ON–OFF (or “telegraph”) model (Ko 1991). The key property of intrinsic noise is that it underlines expression fluctuations that independently affect individual genes. Each gene is subject to random interactions with the transcriptional machinery, and the random interactions at one gene have little impact on the random interactions at other genes in the genome. By contrast, correlated fluctuations of expression in groups of genes, cells, or organisms should be attributed to stochasticity of extrinsic processes such as short- and long-range cell–cell communication and development, including founder cell effects (Elowitz and Leibler 2000).

Intrinsic and extrinsic are relative terms. Changes in expression in one gene due to intrinsic noise alter the expression of other genes in the network, which can lead to changes in neighboring cells and communication with other tissues via hormonal action, and so on. Thus, initial intrinsic stochasticity of one event can propagate through many layers of interactions in multicellular organisms, providing extrinsic perturbations to groups of genes (Figure 1). These interactions are also stochastic, but because they act on group functions in the next layer of interaction, they lead to correlated responses among genes or cells. These layers of gene-centric, cell-centric, and tissue-centric definitions of intrinsic or extrinsic stochasticity can be abstracted further to the organism and even the population level. In general, correlated fluctuations in groups of genes require an extrinsic component relative to individual genes, such that random fluctuations in an upstream event result in coordinated propagation of stochastic variation. Stochasticity of regulatory events in gene networks, heterogeneities in cell size and cell-cycle phase within cell populations, and developmental memory that fixes stochastic events in lineage founder cells are all sources of gene-extrinsic noise using this formal definition. Separating sources of expression variability is complicated. Sherman *et al.* proposed a hybrid model in which individual genes show intrinsic variability, and groups of genes can show a coordinated response to a stochastic input owing to cell-to-cell difference in the efficiency of transcription machinery interactions (Sherman *et al.* 2015). This model combined intrinsic fluctuations acting on individual genes with extrinsic fluctuations acting on individual cells.

**Figure 1:**
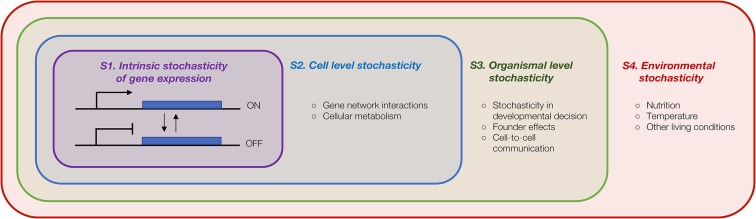
Stochasticity of gene expression at different levels. Stochasticity of gene expression in multicellular organisms can be considered at different level of granularity. Defining intrinsic and extrinsic depends on frame of reference. (S1) From a gene-centric view (purple), stochasticity of biochemical processes defines intrinsic expression stochasticity, with all other processes contributing to external stochasticity. (S2) From a cell-centric view (blue), intrinsic stochasticity also includes stochasticity of the regulatory network, metabolite concentration, and autocrine functions. (S3) On the organism level (green), intrinsic includes development, cell–cell, and tissue–tissue communication. (S4) On the population level (red), stochasticity of the environment is intrinsic. In our work, we measured and modeled gene-level expression noise (A) as influenced by extrinsic factors at the remaining levels.

Gene dose change is an important genomic structural alteration that influences gene expression and phenotype in single-cell organisms. In diploid budding yeast, ∼3% of the genome is haploinsufficient when assayed by growth of deletion mutants in standard rich medium, and much of this effect is due to reduced expression of one-dose genes (Deutschbauer *et al.* 2005). Haploinsufficiency in *Drosophila* appears to be rarer (Lindsley *et al.* 1972; Marygold *et al.* 2007), but it is more difficult to measure subtle differences in fitness in these much larger organisms. At the transcription profile level, the vast majority of gene dose reductions result in an expression phenotype (Malone *et al.* 2012; Lee *et al.* 2016). Mathematically, gene-centric models of gene expression stochasticity indicate that a reduction in gene dose increases intrinsic noise (Cook *et al.* 1998; Bar-Even *et al.* 2006). Given that eukaryotic gene expression occurs in bursts (Raj *et al.* 2006; Pedraza and Paulsson 2008), it follows that expression from two gene copies regulated by independent promoters leads to less expression noise relative to doubled expression from one promoter. It has been proposed that the observed fitness advantage that diploid yeasts have over haploids results from the reduction of expression noise by genome doubling (Wang and Zhang 2011). Understanding the implications of gene dose on expression variability is fundamental for understanding human diseases that originate from DNA copy variants, such as the haploinsufficient developmental disorders associated with many transcription factors (Seidman and Seidman 2002). Indeed, it has been proposed that some human haploinsufficiency syndromes might be related to gene expression stochasticity (Cook *et al.* 1998). For example, haploinsufficiency for the tumor suppressor gene neurofibromin 1 (*NF1*) is accompanied by increased variation of dendrite formation in neurofibromatosis type 1 patients (Kemkemer *et al.* 2002). One attractive explanation of penetrance variability is gene expression stochasticity (Raj *et al.* 2010). Although the effects of dose (with or without dosage compensation) on gene expression variability are conceptually easy to understand at the single-cell level, at the organism level stochasticity of cells in a population should average to mask all evidence of cell-level variation, owing to the central limit theorem. If there is a general link between gene dose and expression variability in complex multicellular organisms, then fluctuations extrinsic to gene expression must be a factor.

In flies, reduction of autosomal gene dose typically results in reduced gene expression, but it is often accompanied by heterogeneous organism-level dosage responses, including increased per-dose expression (dosage compensation) and decreased per-dose expression (expression collapse or anticompensation). Autosomal gene dosage responses are generally locus-specific and propagate in network models, suggesting that feedback provided through biochemical processes and regulatory circuits are causal (Malone *et al.* 2012; Lee *et al.* 2016). The topology of regulatory circuits also influences the way in which expression variability propagates through regulatory networks (Ghosh *et al.* 2005; Alon 2007; Jothi *et al.* 2009; Chalancon *et al.* 2012). Preliminary analysis in *Drosophila* has suggested that reduced autosomal gene dose also increases organism-level expression variability (Lee *et al.* 2016). Thus, noise might also propagate through *Drosophila* gene networks. 

In species with sex chromosomes, there is an interesting wild-type aneuploid state. Flies with two *X* chromosomes are female, and those with one *X* chromosome are males (Erickson and Quintero 2007). Expression of *X*-linked genes in males relative to autosomes is increased approximately twofold relative to the level of each of the two *X* chromosomes in females, thus matching gene dosage between the *X* chromosomes relative to autosomes (Birchler 2016; Kuroda *et al.* 2016). The dosage compensation of *X*-linked genes in male flies is mediated, at least in part, by the male-specific lethal (MSL) complex. The complex activity requires a histone acetyltransferase, males absent on the first (MOF) (Hilfiker *et al.* 1997; Gu *et al.* 1998), which acetylates histone H4 lysine 16 (H4K16ac) within the gene body of transcribing *X* chromosome genes in males (Akhtar and Becker 2000; Smith *et al.* 2000). This specific acetylation event is thought to increase transcription (Gelbart *et al.* 2009; Larschan *et al.* 2011; Conrad *et al.* 2012; Kuroda *et al.* 2016), but there is also evidence that MSL can block expression increases due to this acetylation (Bhadra *et al.* 1999; Pal-Bhadra *et al.* 2005; Prestel *et al.* 2010; Sun *et al.* 2013a,b). MOF is also associated with autosomes as part of a different complex, called non-specific lethal (NSL) (Cai *et al.* 2010; Raja *et al.* 2010), and binds to many housekeeping genes (Feller *et al.* 2012; Lam *et al.* 2012), but does not mediate a consistent effect on the expression of those genes (Zhang *et al.* 2010). Only a subset of NSL-bound genes show a transcriptional effect of MOF (Feller *et al.* 2012), specifically, those with an EBox (also known as Ohler 5 or NDM5) as a core promoter motif (FitzGerald *et al.* 2006; Ohler 2006). Although the influence of MOF on transcription levels has been well studied, the effect of MOF on noise, in the context of either MSL or NSL, has not been explored.

In this study, we examined gene expression variability due to altered gene dose in *Drosophila melanogaster* in multiple different datasets (Chen and Oliver 2015; Lee *et al.* 2016). This involved the analysis of thousands of genes, including most of the major left arm of chromosome 2 (*2L*). We consistently found that the group of autosomal one-dose genes had higher gene expression variability relative to the group of two-dose genes. These results conclusively show that reducing gene dose in *Drosophila* results in increased expression variability at the organism level. Interestingly, autosomal dosage compensation increased expression variability. By modeling, we showed that the difference between expression variability in one-dose and two-dose gene groups that we observed cannot be attributed to the differences in intrinsic gene expression noise alone, but must involve correlated (and thus extrinsic) factors acting on the gene network at the organism level rather than on individual cells.

We also explored the effects of one-dose *X* chromosome genes in wild-type males compared with one-dose genes on the autosomes. In stark contrast with the results for autosomal genes, we found no increase in expression variability for the one-dose genes on the male *X* chromosome. To determine whether this was due to *X*-linage *per se*, we examined *XX* flies using 30 *X* chromosome *Df* lines, and found that one-dose *X* chromosome genes showed autosome-like elevated transcription noise. Thus, the *X* chromosome genes themselves are subject to the same magnitude of stochasticity as autosomal genes. To determine whether reduction in noise is encoded in the male gene expression network, we performed the same analysis of one-dose *X* chromosome genes in *XX* flies transformed from females into males. We observed increased noise in this case as well, suggesting that, as in the case of the autosomes, reduced dose of the *X* chromosome increases noise. Thus, reduced *X* chromosome noise in wild-type males requires dosage compensation, again in contrast to the case of the autosomes. This noise reduction correlates with previously reported MOF binding (Nègre *et al.* 2011), suggesting that MOF is crucial in modulating transcriptional stochasticity. This includes the large number of *X* chromosome genes bound by MSL and the housekeeping genes bound by NSL.

Overall, our study demonstrates that there is increased expression variation of one-dose genes. Although the intrinsic noise due to the physics of kinetics may be an initiator of these fluctuations, the relationship between expression variability and non-MSL dosage compensation indicates a role for network connections and feedback loops, which deliver a response that is coordinated between cells. Therefore, the final organism-level variations are dominated by cell-extrinsic noise. If this is a general property of reducing gene dose, it could contribute to differences in the penetrance of phenotypes in a wide range of organisms. Results on the *X* chromosome indicate that organism-level expression variability can be regulated by evolved pathways.

## Materials and Methods

### Gene expression profiles from RNA-Seq

RNA sequencing (RNA-Seq) analysis of DrosDel (http://www.drosdel.org.uk) deletion flies is described in Lee *et al.* (2016) (for autosomal deletions) and Chen and Oliver (2015) (for *X* chromosome deletions). The results can be also accessed from the Gene Expression Omnibus (GEO) (Barrett *et al.* 2013) under accession GSE61509 for autosomal deletions (2*L*) from pooled whole flies, GSE73920 for autosomal deletions (*2L*) from single whole flies, and GSE60571 for *X* chromosome deletions from pooled heads. We used alignment results from the original studies, where short RNA-Seq reads were mapped on to *Drosophila* genome assembly release 5. We calculated gene-level expression as fragments per kilobase per million mapped reads (FPKM) values with Cufflinks (Trapnell *et al.* 2012) using -G, -b, and -u parameters. Sequencing reads from external spike-ins were not included in Cufflinks analysis to avoid their influence on FPKM measurements. Instead, FPKM values for the spike-ins were separately calculated based on the number of raw reads mapped to the spike-in sequences. We also obtained gene expression fold differences between nondeletion and deletion flies from the original studies.

### Measure of expression variability

To evaluate expression variation, we used RNA-Seq data from two biological replicates. Thus, for each gene in a single deletion experiment, we had two measurements of mRNA levels represented by FPKM values, and we calculated the expression variation metric defined by the absolute difference between two FPKM values divided by their mean:

$$\text{δ}=\frac{2|{\text{FPKM}}_{1}-{\text{FPKM}}_{2}|}{\left({\text{FPKM}}_{1}+{\text{FPKM}}_{2}\right)}$$,

where only genes expressed in both replicates (FPKM_1_ and FPKM_2_ ≥ 0.6829118) were considered. This gene expression cutoff was made based on RNA-Seq signals from intergenic regions. We used the median value of the top 95 percentile of the intergenic signals as described in Lee *et al.* (2016). For just two measurements, δ has a linear relationship with the coefficient of variation (CV), which has been widely used as a metric of cell–cell expression noise:

δ=2CV.

Since δ was based on two replicates only, it cannot be used to measure expression variability of individual genes. However, it can be used to test a relation between two groups of genes, for example, all one-dose genes *vs.* all two-dose genes. As shown in Supplemental Material, Table S1 in File S1, we validated δ for such a test using two different *Drosophila* datasets with a sufficient number of data points to compute the CV. Specifically, we used the dataset of Lin *et al.* (2016b) based on single flies from the *Drosophila* Genetic Reference Panel (DGRP) and our 99 DrosDel lines dataset restricted to chromosomal regions without deletions. Although the Mann–Whitney test based on δ might occasionally be unable to distinguish between two groups of genes that can be distinguished based on CV, the CV and δ values yielded the same false discovery rates for significance thresholds tested in the analysis (Table S2 in File S1). For all expressed one-dose genes in the 99 DrosDel lines, Figures S4 and S5 in File S1 summarize the spread of δ values.

### In silico simulation

The simulation was based on a stochastic model of gene expression, in which formation and decay of single molecules occur randomly (Kaern *et al.* 2005). In the model, genes function independently of each other and can switch spontaneously between repressed and active states with reaction rates *k*_ON_ (activation) and *k*_OFF_ (repression). Active genes are transcribed to mRNA with a constant rate *s*_A_; once a gene is activated, mRNA accumulates until the gene is deactivated. The mRNA degradation rate is δ_M_. All processes are represented by first-order kinetics reactions. Simulations were performed using STOCKS version 1.02 (Kierzek 2002) software for the stochastic kinetic simulation of biochemical processes using the Gillespie algorithm (Gillespie 1977). All simulations started with one or two independent copies of each gene in the repressed state for one- or two-dose genes, respectively. The total number of mRNA copies was reported at each step of every simulation. Each independent simulation represented a time series of changes of mRNA abundance in a single cell generated from the stochastic model. Fluctuations of mRNA copies in single cells from such simulations are attributed only to intrinsic noise.

To model the expression of a single gene in a population of cells, we ran a number of independent simulations (with the same parameters) and computed an average number of mRNA copies for each time point over these simulations. An mRNA-level variation measured by δ value was computed based on two independent runs of such a computation. The computations were done for different cell population sizes. All simulations were repeated to estimate mean and variation of δ values for each cell population size (see Figure 3 and Figure S2 in File S1). In Figure 3, the simulations of one-dose genes were performed for *k*_ON_ = *k*_OFF_ = 0.02/sec (half-time: 35 sec), *s*_A_ = 0.01/s and 0.02/sec for genes without and with compensation, respectively, and δ_M_ = 0.008/sec (half-time: 14 min); in the simulation of two-dose genes, we assumed *s*_A_ = 0.01/sec to get the same expression level as for one-dose genes with compensation. The dependence of expression variation, measured by δ, on promoter rates, transcription rates, and degradation rates is presented in Figure S2 in File S1.

### MOF occupancy

For MOF occupancy we used modENCODE (Nègre *et al.* 2011) data obtained from GEO under accession GSE27806 (modENCODE submission ID 3044), including the assessment of MOF enrichment or depletion. We identified genes that overlapped with the peak regions of MOF occupancy. We then compared the CV of gene expression for genes enriched in MOF occupancy with that for the remaining genes, using the Wilcoxon test. We obtained EBox-motif profiles from a previous study and used peak calls therein (FitzGerald *et al.* 2006). We obtained NSL1 occupancy results (Feller *et al.* 2012) and used the list of NSL-activated genes based on observations from NSL1 knockdown. The *tau* scores were used as described (Lee *et al.* 2016). We considered genes with *tau* scores below the fifth percentile to be housekeeping genes. For all occupancy, motif, and RNA interference results, we updated gene IDs from the original studies to the last annotation of release 5 of the genome [5.57 (McQuilton *et al.* 2012)]. Genes that were merged or split following the update were discarded.

### Data availability

Gene expression profiles appearing in this study can be accessed at GEO with accession numbers GSE61509, GSE73920, and GSE60571. MOF occupancy can be found under GEO accession number GSE27806.

## Results

### Reduction in autosomal gene dosage leads to increased expression variability

To systematically examine the impact of gene dose reduction on expression variability in a multicellular organism, we analyzed gene expression measurements in *D. melanogaster* bearing *Dfs* from the DrosDel collection (Ryder *et al.* 2004, 2007). This collection consists of fly lines that harbor engineered *Dfs* of different chromosomal regions, leaving genes in each line with one dose rather than two.

We used three different sexed DrosDel deficiency line RNA-Seq expression profile sets: (1) pooled whole-fly profiles from 99 different *Dfs* on *2L*; (2) single-fly profiles from 40 different *Dfs* for *2L* (Lee *et al.* 2016); and (3) head profiles from a set of 19 different *X Dfs* and 11 different *3L Dfs* (Chen and Oliver 2015). We used the head expression profiles to address expression variability characteristics of the *X* chromosome in females. In all cases, we only considered genes that were expressed above intergenic background measured as FPKM in each study (Chen and Oliver 2015; Lee *et al.* 2016). The *Dfs* delete ∼40 genes per line, so these studies allowed us to collect thousands of data points on the effects of reduced gene dose. Overall, we measured expression of 4838 one-dose genes from expression profile set 1; 2964 from expression profile set 2; and 1564 from expression profile set 3. To leverage this large number of measurements for assessing differences in expression variation between the group of one-dose genes and the group of two-dose genes, we performed group-wise comparison of replicate-to-replicate expression variation between the groups. This allowed us to bypass the need to estimate expression noise of individual one-dose genes, a task that would require trading the large number of deletion experiments for a large number of replicates of the same experiment. Specifically, to detect differences in expression variation between two groups of genes, we used an absolute difference in expression between replicates normalized by the average of these two values, *i.e.*, δ (*Materials and Methods*). The statistical power comes from the large number of genes assayed.

To unambiguously demonstrate that the statistical difference in δ values between two groups of genes is indicative of statistical difference in expression noise of individual genes in the respective groups, we used an independent experimental dataset where expression of 726 individual flies from the DGRP (Mackay *et al.* 2012) was measured using eight single-fly biological replicates per condition (Lin *et al.* 2016b). Additionally, as the *Dfs* lines are in the same genetic background, we could consider two-dose genes as replicates across lines. Similar analysis of the 99 DrosDel dataset limited to chromosome arms without any deletion also supported the use of statistical difference in δ values as a proxy to measure statistical differences in expression variability between two different groups of genes (Table S1 in File S1).

**Figure 2:**
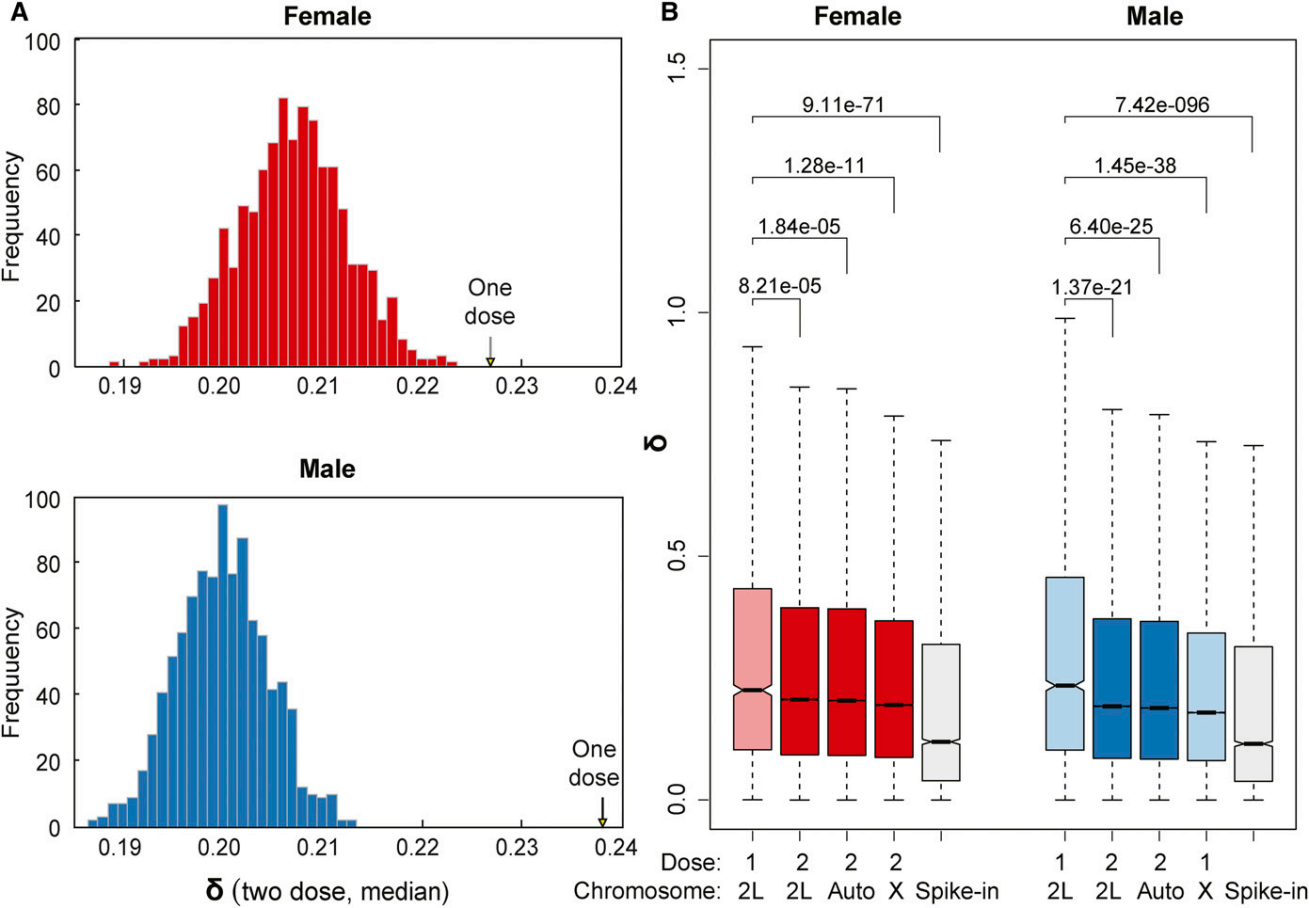
Expression variation of one-dose genes. (A) The median of δ values for all autosomal one-dose genes (arrows) pooled separately from female (above) and male (below) samples is compared with the distribution of median of δ values for the same genes when they are two dose (histogram). (B) Boxplots show the distribution of δ values for one- and two-dose genes on chromosome arm *2L*, other autosomal arms, and the *X* chromosome for each sex, as well as for the spike-in controls from the External RNA Controls Consortium (Jiang *et al.* 2011). The plots show medians (bar), 95% confidence intervals (notch), 25 –75 percentiles (box), and 1.5× interquartile ranges (whisker). Outliers are excluded. *P* values were obtained from Wilcoxon rank sum tests to compare *δ* values of one-dose genes on *2L* *vs.* two-dose genes on *2L*, two-dose genes on other autosomal arms, and genes on the *X*. The same boxplot design and statistical tests have been used for the rest of the figures in this study. Data from the 99 DrosDel set are used here.

To estimate differences in expression variability between groups of one- and two-dose genes, we first compared the median of δ values over all one-dose genes with the distribution of median δ values for the same genes when they were two dose (values from nondeleted segments in other *Df* lines) (Figure 2A). We observed that female gene expression was generally noisier than that of males. This has been previously reported and attributed to sex-biased responses to stochastic changes in the microenvironment (Lin *et al.* 2016a). For both sexes, we observed that one-dose genes had significantly higher δ values than two-dose genes. The results for the 40 *Df* single-fly dataset were qualitatively the same (Figure S1A in File S1). These results demonstrate that populations of one-dose genes show higher expression variation than the exact same genes when they are present in two doses, and highlights sex differences in both one-dose gene responses and overall variance between the sexes.

To look at expression variability in populations of one- and two-dose genes by chromosome, we compared expression variations of all one-dose genes from all *Df* lines to the expression variation of all two-dose genes genome-wide. To control for any long-range effects of the *Dfs* on gene expression, we binned *2L* separately. Given that the entire *X* is one dose in males, we also binned it separately. Finally, we also included analysis of the external spike-in controls to measure technical noise (Lee *et al.* 2016) (Figure 2B). Because RNA-Seq is a sampling technique, genes with poor expression show more measurement variability. We used a stringent low-expression cutoff in our analysis (see *Materials and Methods*). More importantly, all biological expression variability was significantly greater than technical variability. As a result, in no case did we observe a correlation between expression levels and expression variability in groups of genes. We observed the most dramatic and significant increases in δ values among genes with reductions in gene dose due to *Dfs*. Interestingly, this also showed that expression variation for one-dose *X*-linked genes in males was low for these genes that naturally occur in one dose. As in the locus-level results, the populations of one- and two-dose genes in the 40 *Df* dataset were similar (Figure S1B in File S1). Thus, there are fundamental differences between measured expression variability of the one-dose and two-dose genes on *2L*, and between one-dose expression variability on *2L* and on the *X* in males. We will return to the unusual male *X* chromosome response later.

### Organism-level expression variation and cell-extrinsic stochastic processes

**Figure 3:**
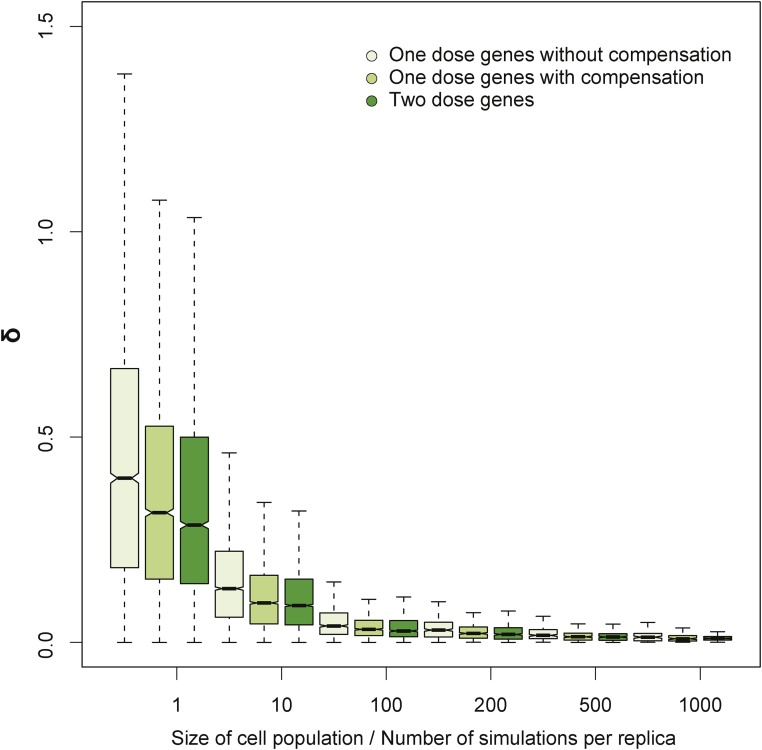
Simulated intrinsic stochasticity of gene expression. We simulated gene expression variability due to intrinsic gene expression noise using the Gillespie algorithm (Gillespie 1977) under the random telegraph model for one-dose genes without and with compensation (light and medium green) and two-dose genes (dark green) for increasing numbers of cells in the population. Mean expression of one-dose genes is reduced by half or the same as expression of two-dose genes, for one-dose genes without and with compensation, respectively.

Theoretical models predict that expression noise in single cells is a function of gene dose (Cook *et al.* 1998). As we mentioned earlier, we thought this was unlikely to be the mechanism in whole-organism assays, owing to the effects of averaging of stochastic events over vast numbers of cells. To determine whether this was the case, we modeled whether, in the context of multicellular organisms, differences originating from single-cell noise will persist or will average out in a large population of cells, and thus drop below detection levels. Specifically, we performed stochastic simulations of intrinsic gene expression variation in cell populations. For these simulations, we followed the definition of intrinsic noise proposed by Elowitz *et al.* (2002). Accordingly, we modeled eukaryotic gene transcription using the broadly accepted random telegraph model (Ko 1991; Raj *et al.* 2006; Pedraza and Paulsson 2008; Larson *et al.* 2009) (*Materials and Methods*). We simulated two equal cell populations to model two biological replicates using a stochastic gene expression model and averaged the results over a given number of cells in each population (Figure 3). We modeled with and without compensation. Although our simulations were based on a particular model of gene expression, conclusions are robust to differences in modeling (*i.e.*, initiation, elongation, and degradation rates; Figure S2 in File S1). Our simulations confirmed that if a population consists of a small number of cells, intrinsic gene expression noise could lead to large expression variation, resulting in large δ values. As expected, these independent single-cell effects quickly averaged out for populations of cells. Consequently, intrinsic noise is vastly exceeded by even low-level technical noise when populations of cells present in an adult fly are measured (Figure 2 and Figure 3).

The above argument generalizes to levels of noise outlined in Figure 1 under a very general assumption formalized below. For example, in a whole-organism measurement, cell-level expression can include stochastic events in transcription of individual genes and the regulatory network within that single cell. By the central limit theorem, as long as we compare populations that are generated according to the same stochastic model, the average expression within each population (of genes, cells, organisms, or groups of organisms) converges to a normal distribution centered at the expected value with variance inversely decreasing with population size. Thus, the differences will average out as the numbers increase. At the same time, extrinsic variations can lead to differences in parameters of the population models, leading to expression differences that can persist in large populations. Therefore, organism-level differences in expression variability of one- and two-dose genes cannot, under these robust assumptions, be attributed exclusively to intrinsic expression variation occurring at the gene level independently within each cell, but rather must require factors extrinsic to individual genes and cells. Likely explanations include developmental noise (including intrinsic noise in founding cells that become epigenetically fixed), perturbation in the microenvironment, or coordinated regulation with different stable states following dose perturbation among organisms.

### Relationship between expression variation and autosomal dosage compensation

**Figure 4:**
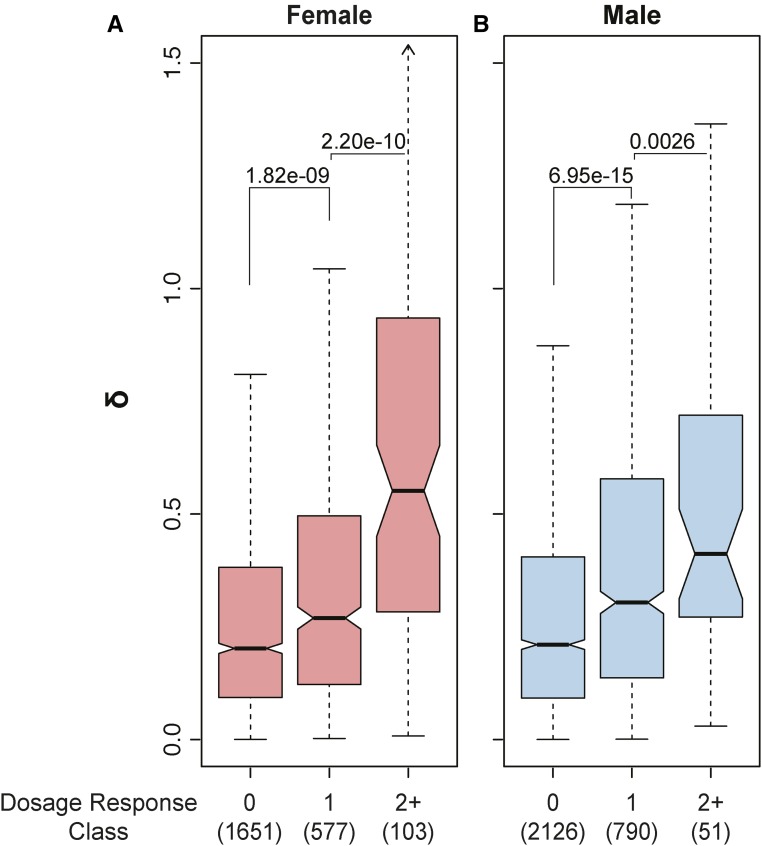
Gene dosage response *vs.* expression variation. Boxplots of expression variability as a function of gene dosage response in female (A) and male (B) flies. Dosage response is defined as | log_2_(*FC* + 1) |, where *FC* is fold change (*Df*/*+* *vs.* control) rounded to the closest integer (see *Materials and Methods*). Numbers in parentheses indicate the number of genes in each bin. Data from the 99 DrosDel set are used here.

The response to reductions in autosomal gene dose is heterogeneous and gene-specific, owing to gene regulatory interactions such as feedback (Malone *et al.* 2012; Lee *et al.* 2016). Similarly, while one-dose genes show, on average, higher variability than two-dose genes, there are broad differences among one-dose genes. Therefore, we asked whether the heterogeneity of dose responses relates to heterogeneity of gene expression variation. To test for a possible relationship between autosomal dosage compensation and expression variation, we considered fold change for all one-dose gene expression relative to two-dose references for those genes. In the case of no compensation, we expect a twofold expression reduction upon deletion of one copy of a gene. This is the one-dose expression baseline in the absence of regulation, and we refer to the variation from this baseline in any direction as a deletion response due to regulation. Positive responses correspond to autosomal dosage compensation, and negative responses correspond to expression collapse, or anticompensation. We use the absolute value of dose responses to capture both types of nonlinear relationships. We found that higher responses to dose also showed higher expression variation (Figure 4). The increased expression variability in genes responding to reduced dose most strongly (*dosage response* ≥ 2) was more pronounced in females than males. We obtained similar results in the 40 *Df* single-fly dataset (Figure S3 in File S1). This shows that the observed expression variation was related to the magnitude of the response to dose change. In general, the response to dosage change implies regulatory feedback through the gene regulatory network. Although gene regulatory networks are extrinsic in relation to the kinetics of transcription of individual genes, these networks exist in individual cells. Therefore, as we outlined previously, when measurements are at the organism level, cells are subject to the same averaging effect as genes. The fact that we observe variance between samples indicates that stochastic regulation has propagated to the organism level.

### Gene copy deletions *vs.* male *X* chromosome genes

Genes on the *X* chromosome have one dose in males and two doses in females. We found that genes on the male *X* chromosome had less expression variation than one-dose autosomal genes (Figure 2B). Remarkably, not only did genes on the male *X* chromosome show less variability than one-dose autosomal genes, but they also showed a statistically significant decrease in variability relative to two-dose autosomal genes. There are three likely mechanisms for the reduced expression variability of one-dose genes on the male *X*. First, the *X* could generally show reduced expression variability owing to evolutionary selection against extrinsic noise susceptibility occuring with each passage of an *X* through males. If this is true, then *X*-linked one-dose genes in *XX* females should also exhibit reduced expression variability. Second, the male gene expression network might be more robust to microenvironmental perturbations, such that decreased expression variability of *X*-linked genes could be due to expression network wiring in males. In this case, genetically transforming an *XX* female bearing a *Df* on the *X* into a male should result in reduced noise for *X*-linked genes. Third, unlike the noise-promoting compensation on the autosomes, the male-specific dosage compensation machinery could reduce expression variability in addition to equalizing the level of gene expression between the *X* and autosomes. In this case, the reduction in noise should occur in *X* males and not in *XX* females transformed into males.

**Figure 5:**
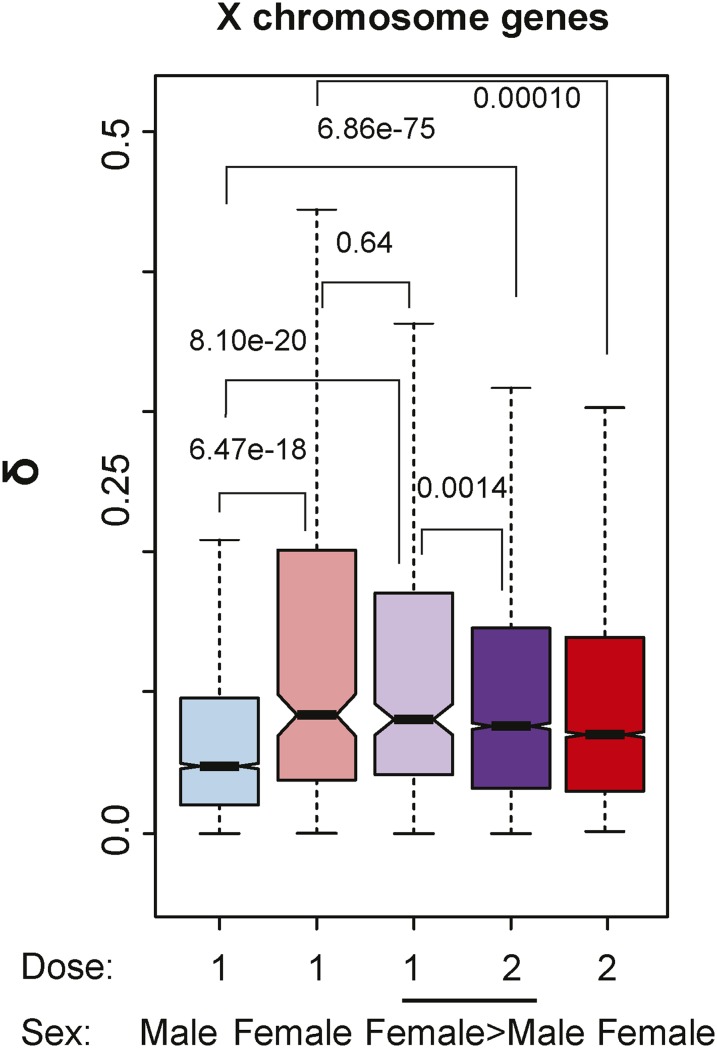
One-dose *X*-linked genes in *Df/+* flies compared with *X*/*Y* males. We compared head expression variation in one-dose *X* chromosome genes from wild-type males (blue), one-dose *X*-linked genes from females (*Df/+*; light red), one-dose (*Df/+*; light purple) and two-dose (purple) genes from females transformed into males (underlined), and two-dose genes from wild-type females (red). The δ value is the mean of three δ values obtained from pairwise measurements among triplicates.

To answer these questions, we utilized expression data from *X* chromosome DrosDel lines, where *XX* flies were female or sex transformed (Chen and Oliver 2015). The expression variation of *X*-linked one-dose genes in *XX* females, or *XX* females transformed into males, showed a significantly higher expression variability compared with one-dose *X*-linked genes in wild-type males (Figure 5). The higher *X* chromosome noise in females or females transformed into males indicates that the *X* is not inherently less noisy than autosomes, and that the male-biased gene expression patterns resulting in a phenotypic male do not reduce noise. Furthermore, the one-dose *X* chromosome genes in wild-type males show less noise than those same genes when in two doses in both females and females transformed into males. These data raise the possibility that the male-specific dosage compensation machinery reduces expression variation.

**Figure 6:**
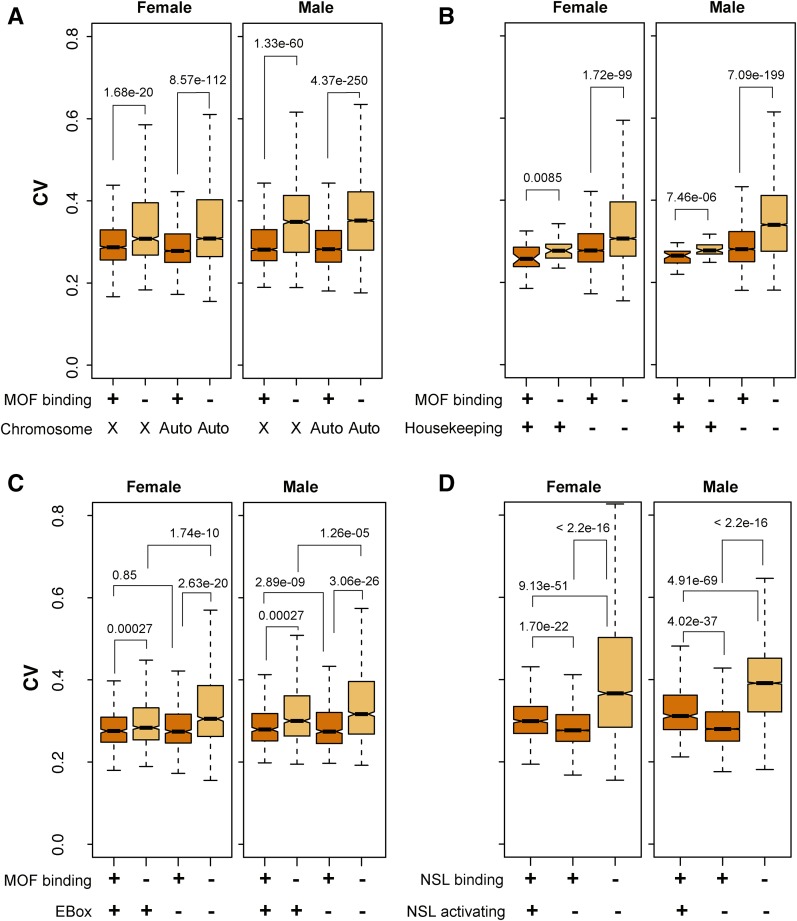
Expression variation and MOF histone acetyltransferase occupancy and activity. Boxplots display expression variation of genes enriched (+, orange) or nonenriched (−, light orange) for MOF or NSL occupancy. Expression variability was assessed by CV. Genes from chromosome *2L* (where the *Dfs* reside) were excluded. (A) Binning by MOF occupancy and *X*-linked (*X*) *vs.* autosomal (auto) genes. (B) Binning by MOF binding and housekeeping *vs.* nonhousekeeping autosomal genes. We identified housekeeping genes based on tissue specificity defined by *tau* (Yanai *et al.* 2005). (C) Binning by MOF binding, and presence (+) or absence (−) of an EBox motif (CAGCTSWW) at the promoter. (D) Binning by NSL1 binding and positive (+) or absent (−) transcriptional responses (“activating”) to NSL1+. MOF or NSL occupancy is indicated in all plots (+, orange; −, light orange). Expression data from the 99 DrosDel lines dataset are used here.

To explore the role of the male-specific dosage compensation systems, we concentrated on MOF, which is a component of the MSL complex that associates with most transcribed *X*-linked genes in males, and a component of NSL at scattered sites in the genome in both sexes. This allowed us to compare *X* and autosome responses. We utilized occupancy data for MOF (Nègre *et al.* 2011) to ask whether this chromatin-modifying machinery might dampen noise. To compare expression variability of genes with and without MOF enrichment, we used data from 99 deletion lines to compute the CV of all expressed genes excluding genes on 2*L* (see *Materials and Methods*) as a function of gene-level enrichment for MOF occupancy. Considering *X*-linked and autosomal genes separately, we then compared the CV values for genes enriched in MOF with the CV values of the remaining genes in the respective group. Genes enriched for MOF occupancy showed reduced expression variability relative to other genes for both the *X*-linked group and autosomes (Figure 6A), consistent with a role for MOF in noise reduction.

### MOF and housekeeping genes

The NSL complex binds and regulates housekeeping genes (Feller *et al.* 2012; Lam *et al.* 2012). Thus, it is possible that reduction in gene expression variability is due to expression-level stability of housekeeping genes rather than MOF function *per se*. To test this possibility, we defined housekeeping genes based on the *tau* score for tissue specificity (Yanai *et al.* 2005), operating under the assumption that housekeeping genes are the most widely expressed. We observed modest correlation between *tau* score and gene expression noise, indicating that housekeeping genes have lower expression noise (Figure 6B). Importantly, MOF binding further reduced expression variability regardless of housekeeping characteristics (*P* < 0.01 for both female and male). This observation suggests that MOF-based reduction of gene expression variability is not due to confounding with housekeeping functions of MOF target genes.

NSL further provides an opportunity to separate noise-dampening roles of MOF from transcriptional effects. NSL activates only a subset of the genes it binds; specifically, those with an EBox motif upstream of transcription start (Feller *et al.* 2012). The reduction of gene expression variability by MOF appears to be independent of quantitative aspects of transcription, as genes with core promoters with or without EBox motifs showed similar reductions in expression variability from both male and female autosomes (*P* > 0.1, Figure 6C). We also compared genes that bind NSL and are activated by NLS *vs.* those with no transcriptional effect (Figure 6D). Expression noise was reduced in both classes. Thus, MOF is likely to minimize gene expression variability even without upregulating gene expression. Our results suggest that, in addition to its proposed roles in *X* chromosome dosage compensation and regulation of a subset of housekeeping genes on the autosomes, MOF and H4K16ac play a part in minimizing potentially harmful expression variation regardless of gene dose.

## Discussion

Measuring gene expression in a set of DrosDel lines, we found that, in *Drosophila*, one-dose autosomal genes show elevated levels of expression variability relative to two-dose genes at the organism level. Where this noise arises is important. Gene expression is subject to intrinsic and extrinsic noise at multiple levels. Computational models indicate that a reduction in gene dose increases intrinsic expression variability of individual genes; however, measurement at the organism level cannot detect this intrinsic expression noise owing to the central limit theorem. The effects of one-dose genes on expression variability at the gene level must average owing to the large numbers of individual cells with intrinsic stochastic behaviors. In agreement, our simulations illustrate that the single-cell fluctuations occurring in each cell independently are irrelevant on the whole-organism scale, where the expression variation is averaged over tens of thousands of cells. Increased expression variability and suppressed noise owing to dosage compensation cannot simply be attributed to intrinsic noise alone as has been previously suggested (Yin *et al.* 2009). Our analysis suggests that observed organism-level expression differences must involve cell-extrinsic perturbations. Although it might sound like an oxymoron, extrinsic noise affecting one-dose genes is coordinated among the cells within a given organism, such that variability in expression responses between organisms can be observed. In more formal language, extrinsic noise perturbs the parameters of stochastic processes within cells. This is reminiscent of incomplete phenotypic penetrance or expressivity due to a given mutant allele, which usually does not occur in individual cells but involves a variable response at the tissue, organ, and organism level. Although the initial triggers might be intrinsic noise in the few founding cells giving rise to a lineage and/or subtle differences in the environment, we suggest that the noise we observe is extrinsic and is due to network interactions at the organism level.

Transcriptional regulation often results in nonlinear responses (Alon 2007). We previously observed that *Drosophila* displays a marked heterogeneous response to gene dose reduction of autosomal genes that propagates through the gene expression network (Malone *et al.* 2012; Lee *et al.* 2016). Many one-dose genes show some dosage compensation via increased gene expression; however, levels of compensation vary drastically for individual genes. Some genes are overexpressed owing to reduced gene dose, also known as the inverse effect (Birchler and Veitia 2012; Sun *et al.* 2013b). Some genes show much more than 50% reduction in expression when dose is reduced by 50%. The different regulatory responses to gene dose reduction in flies may be related to the differences in the need for homeostatic expression levels and/or the design of cell circuitry (*i.e.*, gene regulation) to deliver corrective responses. Our results indicate that dosage responses mediated by such network-based compensatory mechanisms adjust output (Malone *et al.* 2012; Lee *et al.* 2016), while simultaneously decreasing the consistency of gene expression (this study). Thus, for example, when autosomal dosage compensation is present, gene expression variability increases. We can think of autosomal dosage compensation as a process that, via feedback loops and/or other properties of interaction networks, shifts the parameters of the system. The shift itself is noisy. This variation could be the result of multiple possible set-point solutions imposed on one-dose genes, for example. If this noise is a general effect in regulatory networks (Lander 2011), one can easily see how gene dose ultimately leads to phenotypic variability. Indeed, our work complements previous results, which demonstrated that mutations in developmental networks can expose otherwise buffered stochastic variability in gene expression, leading to pronounced phenotypic variation (Becskei and Serrano 2000). Similarly, there are phenotypically heterogeneous responses to copy number variation associated with several complex disorders in humans (Beckmann *et al.* 2007; Wellcome Trust Case Control Consortium *et al.* 2010), including a variety of neuropsychiatric disorders such as autism (Girirajan *et al.* 2011; Poultney *et al.* 2013). Our results suggest that extrinsic expression variability in genes with altered gene dose can contribute to this heterogeneity.

In contrast to the modest overall expression compensation on autosomes (Zhang *et al.* 2010; Malone *et al.* 2012; Lee *et al.* 2014, 2016), *X*-linked genes in males are well compensated (Muller 1932; Straub *et al.* 2005; Gupta *et al.* 2006; Zhang *et al.* 2010; Larschan *et al.* 2011; Conrad *et al.* 2012; Kuroda *et al.* 2016). While we do find differences in expression noise in the sexes as a general trend, the *X* chromosome is exceptional. The low expression variability of one-dose *X*-linked genes in males relative to the same one-dose genes in *XX* females or *XX* females transformed into males (Chen and Oliver 2015) suggests that the reduction in variability is not an inherent property of *X* chromosome genes or reduced variability of the male regulatory network *per se*. This indicates that the sexually dimorphic noise properties of the *X* chromosome occur upstream of the splicing regulator *transformer-2* (*tra2*) that was used to sex-transform the flies. The TRA2 protein, along with the protein produced by the *tra* locus, is required for the sex-specific splicing of the transcription factors encoded by *doublesex* and *fruitless* (Clough and Oliver 2012). The splicing of the *tra pre-mRNA* is regulated by another splicing factor encoded by *Sexlethal* (*Sxl*). The *Sxl* locus is an also an important negative regulator of the MSL component encoded by the *msl2* locus in females (Kuroda *et al.* 2016). In the absence of MSL2, MOF does not preferentially localize to the *X*. By elimination, our data suggest that the MSL complex dampens extrinsic transcriptional noise on the *X*. Thus, in contrast to the variable dosage responses on autosomes and female *X* chromosomes, the dosage compensation of *X* chromosome genes in males, which is a normal state for this organism, has evolved to both increase expression levels relative to autosomes and decrease expression variation. This conclusion holds regardless of numerator or denominator effects of MOF in *X* chromosome dosage compensation relative to autosomes (Birchler 2016; Kuroda *et al.* 2016), since both models are based on increased MOF occupancy on the male *X* chromosome.

Importantly, we also observed that MOF occupancy correlates with reduced noise on the autosomal genes that are associated with another MOF complex, NSL. The activity of MOF on the autosomes has been somewhat enigmatic, as the transcriptional consequences of loss of MOF are minor (Zhang *et al.* 2010) and restricted to a set of housekeeping genes with a particular EBox core promoter element (Feller *et al.* 2012). Our results show that MOF-bound genes with or without the EBox have indiscernible reductions in transcriptional noise. Thus, MOF effects on transcriptional activation and noise reduction appear to be distinct. Given the fact that these MOF-occupied housekeeping genes are broadly and fairly uniformly expressed, we suggest that expression of these genes in a narrow range is important for optimal function, suggesting a new function for NSL and MSL, and the H4K16ac modification that they write. Interestingly, the H4K16ac mark in yeast is removed by the histone deacetylase encoded by *sir2*, and loss of *sir2* reduces gene expression variability at the population level (Anderson *et al.* 2014). This suggests that extrinsic noise suppression by H4K16ac is a widespread phenomenon. How can a gene-level chromatin modification generate a noise-reduction governor at the organism level? In populations of yeast cells, founder cells can epigenetically fix the response of a colony relative to adjacent colonies (Anderson *et al.* 2014). In multicellular organisms like *Drosophila*, one mechanism could be establishment of a chromatin state in a small group of founder cells, and epigenetic transmission of that state to the large groups of genes in the tissues that derive from those founders. There is certainly precedence for this type of memory function for the Polycomb and Trithorax classes of chromatin regulators (Kassis *et al.* 2017). A prediction of this model is that autosomal one-dose genes, especially those subject to regulation in response to dose reduction, should show variable chromatin states from individual to individual. Further, the low noise at genes binding MOF predicts consistent patterns of H4K16ac within and between organisms.

## Supplementary Material

Supplemental material is available online at www.g3journal.org/lookup/suppl/doi:10.1534/g3.117.300400/-/DC1.
