# Supplementary material for: Dosage-Dependent Expression Variation Suppressed on the Drosophila Male X Chromosome
Source: G3 (Bethesda). 2017 Dec 13;8(2):587–98. doi: 10.1534/g3.117.300400 (PMC5919722; doi:10.1534/g3.117.300400)
Supplement: Supplementary file 1 [file 587FileS1.docx]

**Supplemental Materials.**

**Figure S1. Repeat of Fig 1 analysis using an additional deficiency line dataset.** Data from single flies bearing 40 different *Df*s**.** (A) The median of δ values for all one dose genes (dark bar), pooled separately from female (above) and male (below) samples, is compared with the distribution of median of δ values for the same genes when they are two dose (open bars in histogram). (B) Boxplots show the distribution of δ values for one and two dose genes on chromosome arm 2L, other autosomal arms, and the *X* chromosome for each sex. Please refer to Figure 2 for the boxplot representation and statistical test.

**Figure S2. Expression variability models including promoter transition, transcription and degradation rates.** Boxplots represent distributions of simulated expression variation as measured by δ value for increasing cell population size (number of simulations) per replicate in a stochastic gene model. The reference simulation (open bars) was performed using activation and repression rates kON = kOFF = 0.02/s (half-time: 35s), transcription rate s_A_ = 0.02/s and degradation rate δM = 0.008/s (half-time: 14min). The comparison was done to show how the results depended on (A) an increase of promoter transition rates (kON = kOFF increased by 10 and 100 fold), (B) an increase of transcription rate (sA increased by 10 and 100 fold), and (C) a proportional increase of transcription and degradation rates (sA and δM proportionally increased by 10 and 100 fold). Simulations were performed using stochastic kinetic simulation of biochemical processes with the Gillespie algorithm as described in Materials and methods.

**Figure S3. Dosage response vs. gene expression variation in the 40 *Df* line dataset.** Boxplots of expression variability as a function of gene dosage response in female (A) and male (B) flies as in Figure 4.

**Figure S4. Variability of one dose gene expression between two replicates in the 99 *Df* line dataset.** All one dose genes expressed in both replicates are shown (black dots). Our expression variability metric (δ) values are represented by contour lines.

**Figure S5. Relationship between expression level and expression variability in the 99 *Df* line dataset.** For every one dose gene in females and males, average expression level and δ are shown. Pearson’s correlation coefficient and p-values are also given.

**Table S1. The frequency of rejecting the null hypothesis that values of CV or δ for two equally sized random sets of genes come from the same distribution.**

| ***Sex*** | **Female** | | | | **Male** | | | |
| --- | --- | --- | --- | --- | --- | --- | --- | --- |
| ***Significance level*** ^1^ | **0.01** | | **0.001** | | **0.01** | | **0.001** | |
| ***Expression variability measure*** | **CV** | **δ** | **CV** | **δ** | **CV** | **δ** | **CV** | **δ** |
| DGRP dataset ^2^ | 0.01 | 0.01^4^ | 0.001 | 0.001^4^ | 0.01 | 0.01^4^ | 0.0009 | 0.0009^4^ |
| DrosDel dataset ^3^  all genes | 0.009 | 0.011 | 0.0007 | 0.0007 | 0.011 | 0.009 | 0.0011 | 0.0008 |
| DrosDel dataset  500 genes | 0.009 | 0.011 | 0.0012 | 0.0009 | 0.01 | 0.01 | 0.001 | 0.0009 |
| DrosDel dataset  1000 genes | 0.011 | 0.009 | 0.0011 | 0.0016 | 0.01 | 0.009 | 0.0005 | 0.0007 |
| DrosDel dataset  2000 genes | 0.01 | 0.01 | 0.0008 | 0.0011 | 0.011 | 0.011 | 0.0008 | 0.0012 |
| DrosDel dataset  5000 genes | 0.01 | 0.01 | 0.0009 | 0.0006 | 0.011 | 0.01 | 0.0011 | 0.0013 |

^1^ Mann-Whitney test.

^2^ Genes expressed in all replicates from [(Lin, Golovnina, *et al.* 2016)](https://paperpile.com/c/Eotmyw/mMYFl).

^3^ Two dose autosomal genes only.

^4^ δ was computed based on two randomly selected flies.

**Table S2. Median *p* values as well as fraction of *p* > 0.01 incidents between δ values from two equally sized subsets of genes with high and low CV values.**

|  | **Female** | | **Male** | | |
| --- | --- | --- | --- | --- | --- |
|  | **Median *p* value** ^1^ | **Fraction of**  ***p* ≥ 0.01**^1^ | **Median *p* value** ^1^ | | **Fraction of**  ***p* ≥ 0.01**^1^ |
| DrosDel dataset ‐ 500 genes | 1.7E­‐09 | 0.0001 | 9.3E-08 | 0.0015 | |
| DrosDel dataset ‐ 1000 genes | 2.6E­‐18 | 0 | 2.4E-­16 | 0 | |
| DrosDel dataset ‐ 2000 genes | 4.4E­‐33 | 0 | 2.3E-­28 | 0 | |
| DrosDel dataset - 5000 genes | 4.0E­‐82 | 0 | 1.2E-­67 | 0 | |

^1^ Mann-Whitney test
